# A novel synthetic peptide microarray assay detects *Chlamydia* species-specific antibodies in animal and human sera

**DOI:** 10.1038/s41598-018-23118-7

**Published:** 2018-03-16

**Authors:** Konrad Sachse, Kh. Shamsur Rahman, Christiane Schnee, Elke Müller, Madlen Peisker, Thomas Schumacher, Evelyn Schubert, Anke Ruettger, Bernhard Kaltenboeck, Ralf Ehricht

**Affiliations:** 1 Institute of Molecular Pathogenesis, Friedrich-Loeffler-Institut (Federal Research Institute for Animal Health), Jena, Germany; 2 Department of Pathobiology, College of Veterinary Medicine, Auburn University, Auburn, AL USA; 3 Alere Technologies GmbH, Jena, Germany; 4 InfectoGnostics Research Campus, Jena, Germany; 5 Institut Virion\Serion GmbH, Würzburg, Germany; 6 Present Address: RNA Bioinformatics and High-Throughput Analysis, Faculty of Mathematics and Computer Science, Friedrich-Schiller-Universität, Leutragraben 1, 07743 Jena, Germany

## Abstract

Serological analysis of *Chlamydia (C.)* spp. infections is still mainly based on micro-immunofluorescence and ELISA. To overcome the limitations of conventional serology, we have designed a novel microarray carrying 52 synthetic peptides representing B-cell epitopes from immunodominant proteins of all 11 chlamydial species. The new assay has been validated using monospecific mouse hyperimmune sera. Subsequently, serum samples from cattle, sheep and humans with a known history of chlamydial infection were examined. For instance, the specific humoral response of sheep to treatment with a *C. abortus* vaccine has been visualized against a background of *C. pecorum* carriership. In samples from humans, dual infection with *C. trachomatis* and *C. pneumoniae* could be demonstrated. The experiments revealed that the peptide microarray assay was capable of simultaneously identifying specific antibodies to each *Chlamydia* spp. The actual assay represents an open platform test that can be complemented through future advances in *Chlamydia* proteome research. The concept of the highly parallel multi-antigen microarray proven in this study has the potential to enhance our understanding of antibody responses by defining not only a single quantitative response, but also the pattern of this response. The added value of using peptide antigens will consist in unprecedented serodiagnostic specificity.

## Introduction

Detection of antibodies in human and animal infections provides important information to confirm clinical disease, the presence or absence of infection, or determine the immune response after vaccination. While DNA-based diagnostic technology has made remarkable progress in the past decades, serological analysis of microbial infections is still mainly based on the conventional enzyme immunoassays. This applies in particular to infections by *Chlamydia* spp., where the lack of species-specific antibody assays has been a major obstacle to the elucidation of host immune response mechanisms^1^. The family *Chlamydiaceae* with its single genus *Chlamydia* currently comprises 11 species^2^, among which *Chlamydia (C.) trachomatis* (genital disorders, trachoma), *C. pneumoniae* (respiratory disease) and *C. psittaci* (atypical pneumonia) figure as human pathogens. Besides, *C. psittaci* (avian chlamydiosis), *C. abortus* (ovine enzootic abortion), and *C. pecorum* (intestinal, respiratory and urogenital disorders) are economically important infectious agents in domestic animals.

One of the major impediments to highly specific assays is the unique biphasic developmental cycle of these obligate intracellular bacteria. In conventional diagnostic technology, the preparation of host cell-free antigen of *Chlamydia* spp. already requires special expertise and equipment. But even the use of purified whole-cell chlamydial antigen for antibody capture usually implies cross-reactions with other chlamydiae due to close structural similarity among some of the major surface antigens, so that strict specificity at species level is not attained.

Further difficulties in developing reliable antibody detection tests result from the epidemiology of chlamydial infections, which show a wide range of clinical manifestations from acute to symptomless. Latent infections are particularly widespread in humans and animals. Based on a survey in cattle, Kaltenboeck *et al*.^3^ postulated the ubiquitous dissemination of certain endemic *Chlamydia* spp. in large herds of high-density host populations of agricultural production animals and, as a consequence, the difficulty in finding truly negative populations^4–6^.

The micro-immunofluorescence (MIF) test is still regarded as the standard serological assay for species-specific detection of chlamydial antibodies^7^. Poor sensitivity and cross-reactivity were reported^8–10^, which may, however, not be properties of the test but rather reflect the nature of infection and experience of laboratory staff. In addition, a number of custom-made and commercial ELISAs with different specificity levels are being used^11^, but their performance parameters are only satisfactory when well-defined capture antigens are used, e.g. recombinant proteins^12^. In contrast, chlamydial whole-cell preparations as used in many ELISAs and MIF preclude high specificity^1^.

The idea of using synthetic peptides derived from immunological determinants to specifically capture cognate antibodies emerged more than a decade ago^13–15^. A number of studies investigating antibody responses to bacterial^16,17^, viral^18–21^ and parasite^22–24^ infections in humans and animals have been published. In *Chlamydia* research, the major outer membrane protein OmpA or MOMP was the first antigen molecule to be systematically investigated for its immunogenic capacity, and peptides derived from its epitopes were used as capture antigens in a number of studies. Several groups conducted epitope mapping studies of OmpA and tested B-cell epitope-derived oligopeptides sized 6 to 10 amino acids (aa) in serological assays^1,25–28^. While initially providing some promising results, the methodological approach did not find the broad acceptance among the diagnostic community that it deserved. This was probably due to limitations in test performance, particularly low sensitivity^28,29^, and the perception of high cost of peptide synthesis. Meanwhile, advances in proteomics research have qualified the previously assumed immunodominance of OmpA and revealed substantial contributions to humoral immune response of other important chlamydial proteins, such as inclusion protein IncA and polymorphic outer membrane proteins POMPs^30–32^. In this situation, a fresh attempt to develop a peptide-based serological assay could make sense, as our knowledge on immunogenic chlamydial proteins has extended and, in addition, state-of-the-art microarray technology is now available.

Concerning the design of capture peptides, recent investigations^33,34^ highlighted the crucial importance of two parameters: (i) Taking into account that the probability of high-affinity binding between antigen and antibody is proportional to capture peptide length, the optimal size is 16 to 30 aa to cover both linear and the majority of conformational epitopes. (ii) A suitable spacer sequence between solid support and peptide should be introduced to minimize steric hindrance to antibody binding. Although numerous bioinformatic tools for identification of potential B-cell epitopes from protein sequence databases are available, their performance is generally not satisfactory when it comes to experimental verification^34–36^. An extensive comparative study of more than 100 different methodologies revealed that B-cell epitope prediction algorithms from the literature typically lacked the necessary accuracy, whereas scales designed for prediction of protein properties, particularly disorder tendency, identified B-cell epitopes far more reliably^33^.

In the present proof-of-concept study, we have designed and produced a novel microarray carrying a panel of 52 covalently immobilized *Chlamydia* epitope-derived synthetic peptides and experimentally validated it using characterized serum panels from animals and humans.

## Results

### Validation of peptide microarray specificity using murine hyperimmune sera

**Figure 1:**
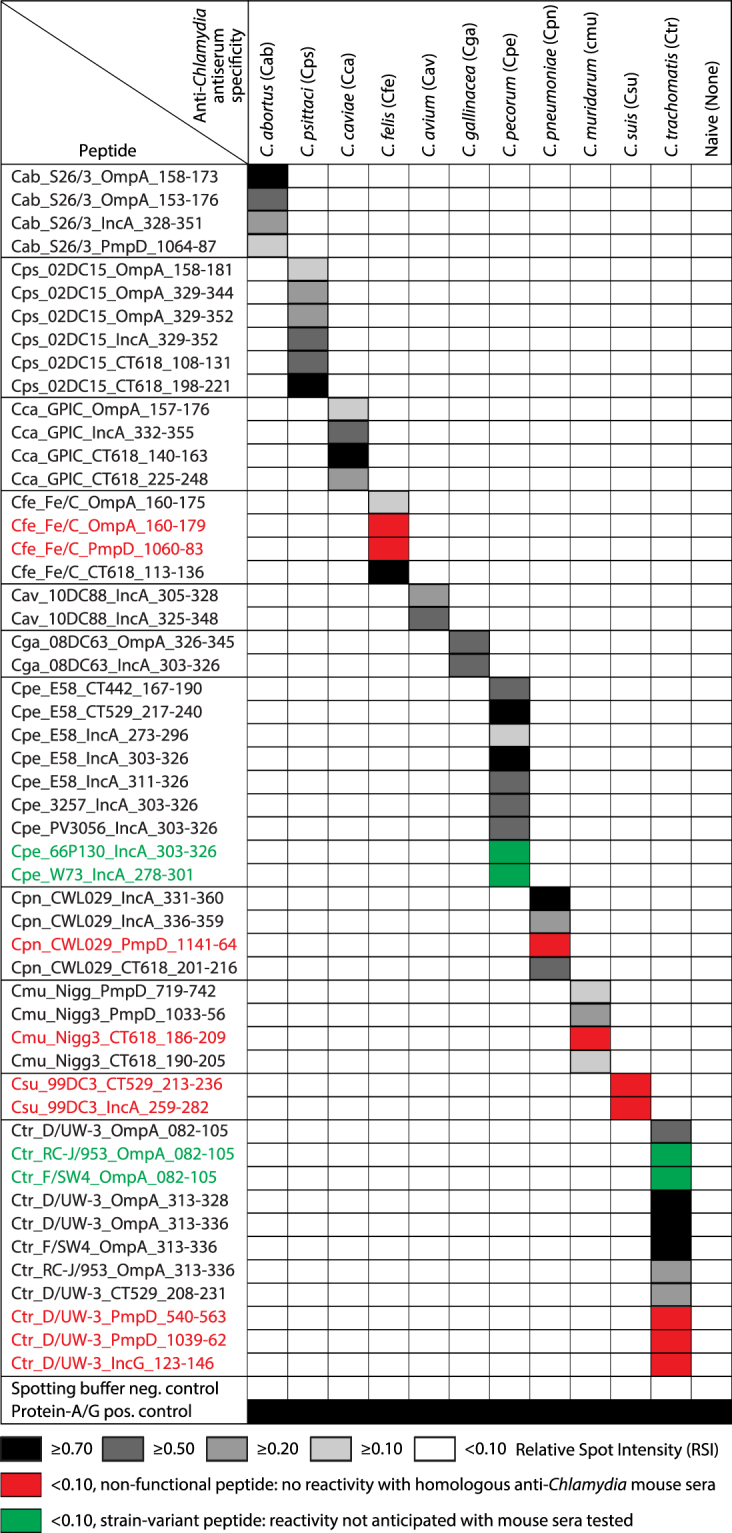
Functionality of ELISA-identified peptide antigens in the peptide microarray. Reactivity of peptide antigens of 11 *Chlamydia* species with homologous, heterologous and naïve pooled mouse sera are shown. All peptide antigens had been previously found reactive by microtiter plate ELIASs using the homologous mouse sera^34^. Peptides were named by three-letter *Chlamydia* species acronyms and the strain, followed by source protein and the amino acid positions of the peptide in the protein. Peptides for the microarray format were synthesized with N-terminal KKK (lysine-lysine-lysine), Ttds (trioxatridecan-succinamic acid) and SGSG (serine-glycine-serine-glycine) linkers followed by the specific sequence and G (glycine-COOH), and covalently linked to the 3-D epoxy chip surface. Each peptide was spotted 2–3 times per microarray, and each microarray was tested 2–3 times with identical sera. An average relative spot intensity (RSI) of ≥0.10 for all spots was used as parameter for functionality (reactivity) of each peptide antigen in the microarray. Abbreviations: *C. Chlamydia*, Cab *Chlamydia abortus*, Cav *Chlamydia avium*, Cca *Chlamydia caviae*, Cfe *Chlamydia felis*, Cga *Chlamydia gallinacea*, Cmu *Chlamydia muridarum*, Cpe *Chlamydia pecorum*, Cpn*, Chlamydia pneumoniae*, Cps *Chlamydia psittaci*, Csu *Chlamydia suis*, Ctr *Chlamydia trachomatis*.

Pooled sera from mice immunized against each of the 11 different *Chlamydia* spp. and two serum pools of naïve mice, all of them previously characterized by ELISA^34^, were examined using the peptide microarray protocol. The functionality of individual peptide substances is illustrated in Fig. 1. The majority of the peptides (39/48) reacted with the homologous *Chlamydia* species-specific pooled sera. Four additional serovariant *C. pecorum* and *C. trachomatis* peptides were included for later use with animal and human sera. As expected, these four peptides did not react with mouse sera because the mice had been immunized with other serovariants of *C. trachomatis* and *C. pecorum*. Importantly, none of the peptides tested showed reactivity with the heterologous or naïve sera, thus indicating 100% specificity. However, 9 of 48 spotted peptides (19%) did not react with the homologous mouse sera (red squares in Fig. 1) although biotinylated peptide antigens from these epitopes were strongly reactive with the same sera when used in microtiter ELISA format. This indicates 81% successful transfer to microarray of peptide antigens identified in ELISA. The transfer failures may be due to differences in peptide antigen length (Supplementary Table S1), or to masking of antibody accessibility by aberrant binding of reactive amino acid side chains.

**Figure 2:**
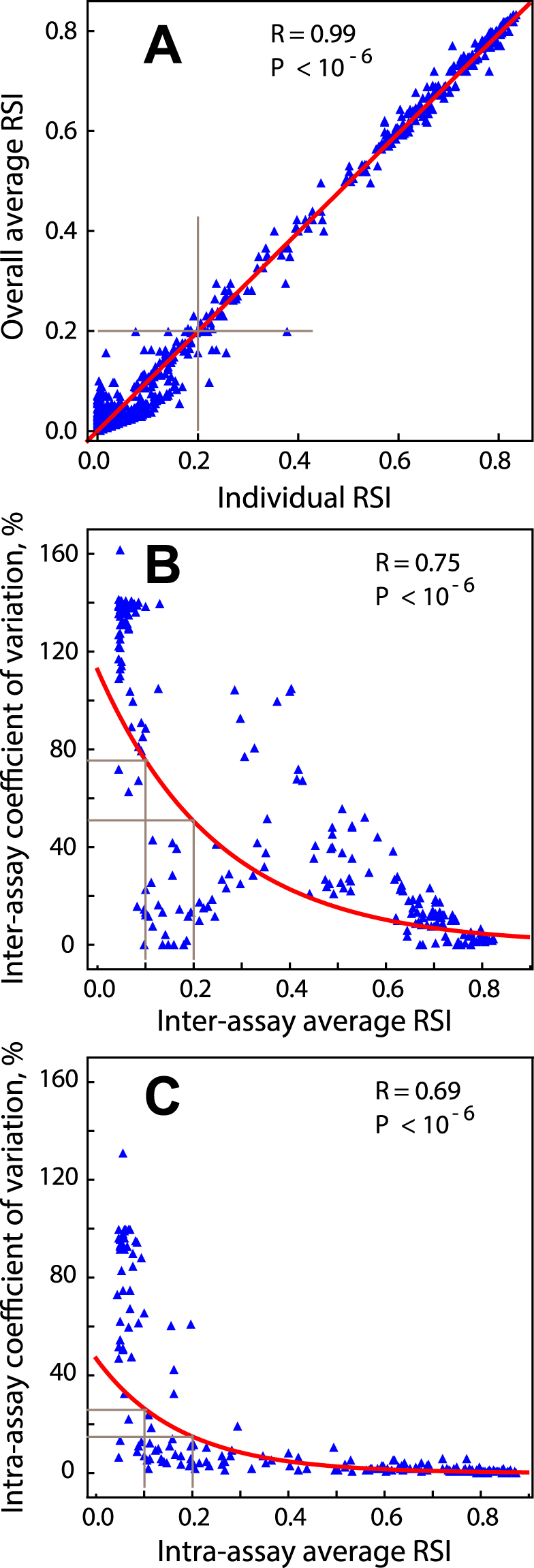
Reproducibility of microarray results evaluated by coefficient of variation. Each peptide was spotted 2–3 times on a chip for intra-assay readings, and each array was tested 2–3 times for inter-assay readings. The overall average of 4–9 intra- and inter-assay signals were used to assess functional peptide antigens in the microarray as shown in Fig. 1. (**A**) All individual readings are plotted versus their corresponding averages, and the 0.2 cut-off is shown that separates weak signals with high variance from strong signals with low variance in intra- and inter-assays. (**B**,**C**) The means in intra- and inter-assays are plotted versus their corresponding coefficient of variation (CV).

The data of the experimental test series have also been used to characterize the reproducibility of microarray results. As can be seen from Fig. 2, individual readings in intra- and inter-assays are very consistent (minimum dispersion) at signal intensities ≥0.2 (relative spot intensity, RSI). In addition, the 10–20% intra-assay coefficient of variation (CV) is lower than the inter-assay CV, indicating that the microarray signal recall is very good. In contrast, the relatively high inter-assay CV possibly reflects the fact that different individuals conducted the experiments in two different laboratories and different production batches of microarrays were used.

### Examination of sera from naturally infected cattle

To explore the peptide microarray’s performance on field samples, 15 sera from calves and cows of well-documented *Chlamydia* infection status^37,38^ were examined.

**Figure 3:**
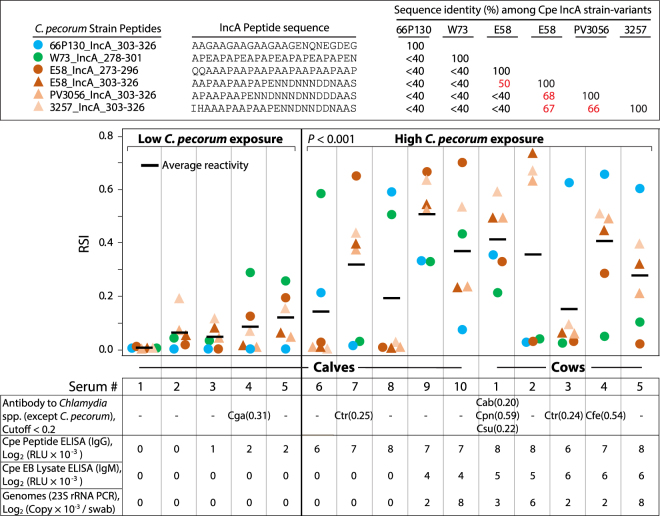
Microarray evaluation with sera from cows and calves with *C. pecorum* infections. Six IncA (Inclusion membrane protein A) peptides of five *C. pecorum* strains are shown in the top panel along with percent sequence identities among the peptide sequences. Sequence identities of 50% or more are shown in red font to indicate potential for cross-reactivity. These C-terminus IncA peptides were chosen to cover known sequence variants in *C. pecorum*^55^. Reactivity of the 6 IncA peptides with 10 calf and 5 cow sera in the microarray assay are shown in the middle panel. Reactivity of the sera with peptides from any other chlamydial species is indicated in the bottom panel. Also shown separately in the bottom panel are 5 of the calves that were 0 to 53 days old and had been less exposed to *C. pecorum* than 5 older calves and the 5 cows, consistent with IgG and IgM antibody levels against *C. pecorum*- peptide and elementary body (EB) lysate ELISAs, and by *Chlamydia* 23SrRNA gene PCR. The difference in average anti-peptide IgG antibody levels between low and highly exposed animals is highly significant (*P* < 0.001, Mann Whitney U test). The 7 PCR-positive calves and cows infected with *C. pecorum* were also confirmed with *ompA* gene sequencing^37,38^.

Figure 3 illustrates that the sera of cows and *Chlamydia*-exposed >7 week-old calves produced significantly higher microarray signals than sera of non-exposed 1–5 week-old calves (P < 0.001, Mann-Whitney U test). This is in line with data from qPCR and antibody ELISAs against *C. pecorum* peptide and elementary body lysate antigens, and fully coincides with the samples’ history as the cows were highly and continuously exposed to *C. pecorum*, whereas the calves were kept separate and their exposure to chlamydiae had just begun^37^.

Data in Fig. 3 and Supplementary Table S2, as well as sequencing of PCR products, indicate that *C. pecorum* was the predominant chlamydial agent in these animals. All six IncA peptides covering major strain-specific sequence variations of *C. pecorum* were immunoreactive, and all of them bound cognate anti-*C. pecorum* antibodies in at least seven sera out of 15 samples tested (Table S2 and Fig. 3). Importantly, the IncA peptides showed strong reactivities with sera of 10 calves and cows highly exposed to *C. pecorum*, but weak or no reactivity with sera of 5 calves with low *C. pecorum* exposure (Fig. 3). In contrast, the two CT442 and CT529 peptides showed reactivity only with a single calf serum (seventh sample; Table S2). Interestingly, the 66P130 and W73 peptides did not react with E58-specific mouse sera (Fig. 1), but, as *C. pecorum* infections with multiple strains were circulating in the herd, several cows showed an antibody response against these epitopes, so that the 66P130 and W73 peptides proved suitable for serological analysis.

Apart from this, one or two lower-intensity signals with peptides of other chlamydial species, i.e. C*. gallinacea, C. trachomatis, C. abortus, C pneumoniae*, or *C. suis*, were observed in 8 of the 15 sera (Supplementary Table S2). Reactivity of *C. trachomatis* peptides probably indicates the presence of antibodies against *C. suis*, because the latter is very diverse in terms of antigenicity (yet poorly investigated because a fully-assembled reference genome was published only recently) and generally closely related to *C. trachomatis*. The significance of these reactions and the issues underlying presumed seronegativity to chlamydiae will be discussed further below.

### Examination of sera from sheep with known vaccination status

**Figure 4:**
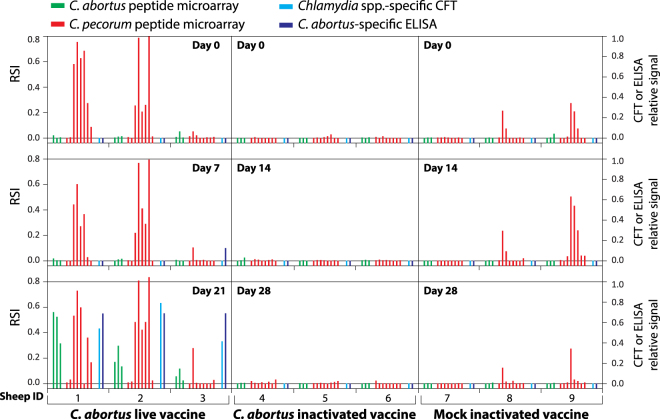
*C*. *abortus*-specific antibodies in sheep before and after *C. abortus* vaccinations. The reactivities of 3 *C. abortus* and 8 *C. pecorum* peptides with sheep sera collected before and after vaccination are shown individually for each animal and time point. Peptides used were Cab_S26/3_OmpA_153-176, Cab_S26/3_IncA_328-351, Cab_S26/3_PmpD_1064-1087, Cpe_66P130_IncA_303-326, Cpe_W73_IncA_278-301, Cpe_E58_IncA_273-296, Cpe_E58_IncA_303-326, Cpe_PV3056_IncA_303-326, Cpe_3257_IncA_303-326, Cpe_E58_CT529_217-240, and Cpe_E58_CT442_167-190. The difference in anti-*C. abortus* peptide antibody levels of sheep 1-3 on days 0 and 7 to day 21 is highly significant (*P* = 0.003, Fisher exact two-tailed test).

A total of 27 sera from three animals vaccinated with a live vaccine, three animals treated with an inactivated vaccine and three mock-vaccinated controls collected at three different time points have been tested using the peptide microarray (Fig. 4 and Supplementary Table S3). *C. abortus* peptide reactivity proved very consistent at the three sampling times on and after *C. abortus* vaccination, while the inactivated vaccine did not generate specific antibodies. Interestingly, the *C. pecorum* microarray peptides consistently detected antibodies in 5 out of 9 sheep pre-and post-vaccination. In contrast, the *C. abortus* peptides reacted only 3 weeks after vaccination with live *C. abortus*, consistent with the highly specific, competitive *C. abortus* ELISA test. Similarly, the *Chlamydia* genus-specific CFT detected antibodies only after live vaccination. However, the *Chlamydia* genus-specific CFT failed to detect long-term antibodies against *C. pecorum*, presumably due to the well-known preferential detection of early antibody responses against chlamydial LPS, the major antigen of the CFT^1,39^.

**Figure 5:**
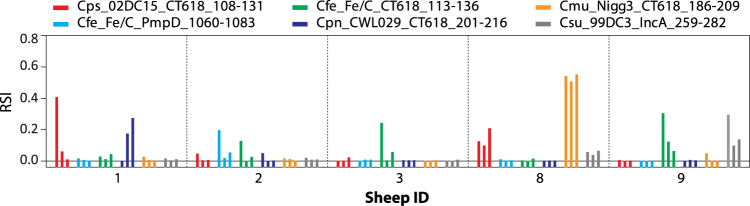
Infrequent presence of antibodies against other *Chlamydia* spp. in sheep sera. Six peptides from 5 chlamydial species showed microarray signals above the background level with 5 of the 9 sheep sera shown in Fig. 4 (one peptide each from *C. psittaci*, *C. pneumoniae*, *C. muridarum* and *C. suis*, and two peptides from *C. felis*). The signal at the 3 sampling time points is indicated by 3 consecutive bars of the same colour.

The microarray identified additional chlamydial antibodies in the *C. pecorum* antibody-positive animals 1, 2, 3, 8, and 9, and occasional reactive signals with other *Chlamydia* spp. in five animals as illustrated in Fig. 5. However, the antibody levels for the non-endemic chlamydiae were typically low (except *C. muridarum* in animal 8), thus either indicating the transient nature of these infections or possibly weak cross-reactivities. In contrast, antibodies against *C. pecorum*, when present, were high as shown in Fig. 4.

### Examination of human sera

**Figure 6:**
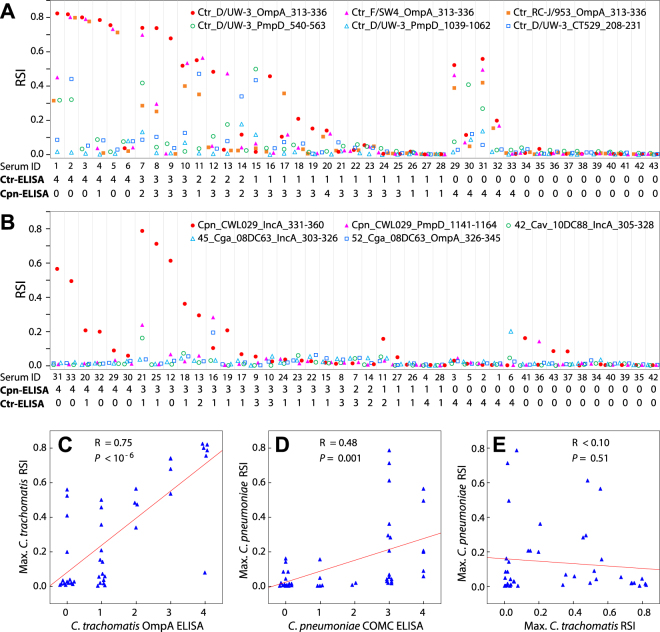
Detection of human exposure to *C. trachomatis* and *C. pneumoniae*. (**A**) *C. trachomatis*-specific antibody detection by use of the strongly reactive outer membrane protein A (OmpA), PmpD, and CT529 peptides. Three strain-variant peptides of OmpA variable domain IV, two peptides of two separate regions from polymorphic membrane protein D (PmpD), and one peptide of protein CT529 (CT_529 locus of *C. trachomatis* D/UW-3/Cx) were used to determine *C. trachomatis*-specific antibodies from 43 serum samples. Separately, in an ELISA with recombinant *C. trachomatis* OmpA antigen, anti-*C. trachomatis* antibody levels of these 43 sera were determined, as displayed below the abscissa where 4, 3, 2, 1, or 0 indicates very strong, strong, moderate, weak, or absent ELISA signal. ELISA signals 4-3 are classified as positive, 2 as borderline, and 1 (above background) and 0 (background signal) as negative. (**B**) *C. pneumoniae*- and remaining *Chlamydia* spp.-specific antibody detection. The strongest microarray signals with an IncA peptide and a PmpD peptide of *C. pneumoniae* are shown. Among the remaining 9 *Chlamydia* spp., three *C. avium* and *C. gallinacea* peptides produced signals with three individual sera. All peptides of the remaining 7 *Chlamydia* spp. produced <0.1 signals in the microarray and are not shown. In a *C. pneumoniae* ELISA with an antigen of complexes of outer membrane proteins (COMC), antibody levels of these 43 sera against *C. pneumoniae* were determined. (**C**) Correlation between *C. trachomatis* OmpA ELISA signals and *C. trachomatis* microarray signals. (**D**) Correlation between *C. pneumoniae* COMC ELISA signals and *C. pneumoniae* microarray signals. (**E**) Correlation between *C. trachomatis* microarray signals and *C. pneumoniae* microarray signals.

A panel of 43 sera from individuals with known chlamydial infection status has been tested with the peptide microarray assay and in-house ELISAs specific for *C. trachomatis* and *C. pneumoniae*, respectively. Specific *C. trachomatis* antibody detection by microarray was mainly based on the strongly reactive OmpA, PmpD and CT529 peptides, i.e. three serovar-variant peptides of the OmpA variable domain IV, two peptides of two PmpD regions, and one CT529-derived peptide (Fig. 6A, Supplementary Table S4). In the case *of C. pneumoniae*, the strongest microarray signals were obtained with an IncA inclusion membrane protein-derived and a PmpD peptide (Fig. 6B, Supplementary Table S4).

**Figure 7:**
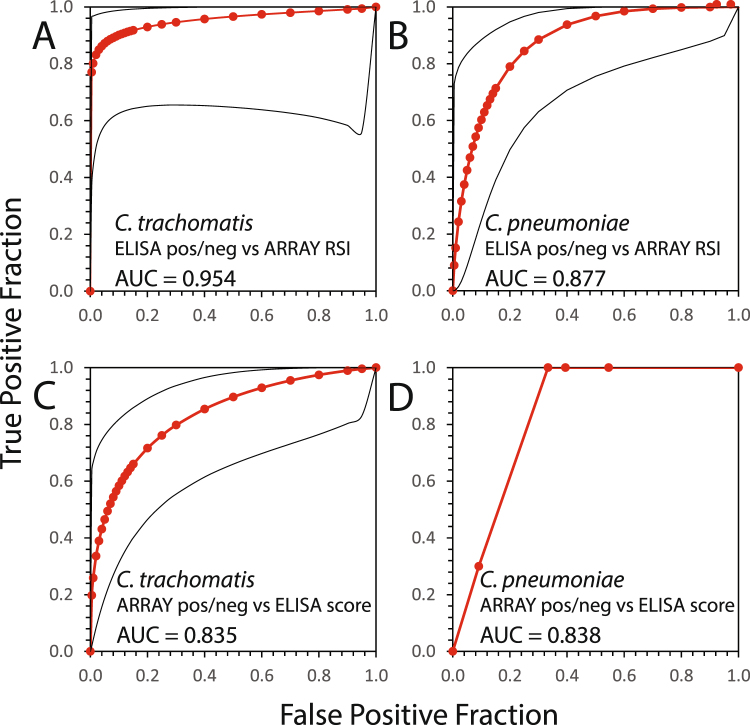
Determination of peptide microarray performance using ROC analysis. The data displayed in Fig. 6, panels A and B, were used to determine performance characteristics of the *C. trachomatis* and *C. pneumoniae* microarray peptides for antibody detection against *C. trachomatis* or *C. pneumoniae* in human sera determined by receiver operating characteristic (ROC) curves and area under curve (AUC). Red lines indicate binormal ROC curves derived by maximum likelihood estimation, black lines indicate 95% confidence intervals. (**A**) ROC analysis of continuous RSI results of *C. trachomatis*-specific antibody detection with OmpA, PmpD, and CT529 peptides in comparison to positive/negative scoring by anti-*C. trachomatis* ELISA. (**B**) ROC analysis of *C. pneumoniae*-specific antibody detection with IncA and PmpD peptides in comparison to positive/negative scoring by anti-*C. pneumoniae* ELISA. (**C**) ROC analysis of ordinal *C. trachomatis* ELISA results in comparison to positive/negative scoring by anti-*C. trachomatis* RSI data with a negative RSI cut-off of <0.2 RSI. (**D**) ROC analysis of ordinal *C. pneumoniae* ELISA results in comparison to positive/negative scoring by anti-*C. pneumoniae* RSI data with <0.2 RSI cut-off. Data for this panel are plotted directly since a binormal ROC curve could not be constructed due to an implied exact-fit that is vertical at constant FPF = 0.091.

At a 0.2 microarray RSI cut-off, the concordance between *C. trachomatis* ELISA and microarray was high (81%), with 8 of the 43 sera giving discordant results (Fig. 6A), and *C. trachomatis* peptide reactivity on the microarray strongly correlated with the OmpA-based ELISA antibody titre (R = 0.75, P < 10^−6^; Fig. 6C). These discordant results are exclusively caused by positive microarray signals for 8 sera that were negative in the *C. trachomatis* ELISA, indicating higher sensitivity of the *C. trachomatis* peptide microarray. Accordingly, by determining a much higher 0.53 RSI cut-off, receiver operating characteristic (ROC) curve analysis found an excellent performance of the microarray, with a 0.954 area under the curve (AUC) driven by 40 correct calls, and only a single false-negative and two false-positive calls (Fig. 7A).

Overall concordance between *C. pneumoniae* ELISA and microarray at 0.2 RSI cut-off was lower (77%). Here, discordant results are exclusively caused by negative microarray signals for 11 sera that were positive in the *C. pneumoniae* ELISA. Similarly, the correlation between ELISA and microarray was weak for *C. pneumoniae* (R = 0.48, *P* = 0.001; Fig. 6D).

Interestingly, ROC analysis determined a similar 0.19 RSI cut-off and found 0.877 AUC driven by ten false-positive calls out of the 43 sera (Fig. 7B).

In a reversal of ROC analysis, we also determined ELISA performance relative to peptide microarray by comparing the ordinal ELISA scores, with borderline scores assumed positive, to positive/negative microarray calls for both *C. trachomatis* and *C. pneumoniae* antibodies using the 0.2 RSI microarray cut-off. This comparison indicated poor *C. trachomatis* ELISA performance with 0.835 AUC, with 1 false-positive and 7 false-negative calls, (Fig. 7C), indicating 96% specificity, but only 65% sensitivity relative to the peptide microarray. *C. pneumoniae* ELISA performance was similarly poor at 0.838 AUC and 13 false-positive calls (Fig. 7D), indicating 100% sensitivity, but only 61% specificity for the *C. pneumoniae* ELISA.

As expected, *C. trachomatis* and *C. pneumoniae* peptide microarray signals did not correlate (*P* = 0.51; Fig. 6E). Human antibody responses to the remaining 9 *Chlamydia* spp. were almost absent except that three peptides of *C. avium* and *C. gallinacea* showed marginal reactivities with only three individual sera out of 43 human sera tested here.

Altogether, the present data confirm the specificity of the microarray and, at the same time, provide evidence on the presence of antibodies to both *C. trachomatis* and *C. pneumoniae* in the same individual, as seen in 9/43(20.9%) serum samples (Supplementary Table S4). It is important to note that this finding is not due to cross-reaction.

## Discussion

The present system represents a versatile platform for serological analysis of animal and human sera and has been demonstrated to detect specific IgG antibodies to 11 different *Chlamydia* spp. The assay protocol is applicable to all mammalian sera for using HRP-conjugated Protein A/G as secondary antiserum. In the case of avian sera, the latter needs to be replaced with an IgY conjugate. With a dynamic range covering five orders of magnitude^40^, the present assay is semi-quantitative, but more precise quantification will be possible by replacing precipitation staining with fluorescence detection.

In the initial evaluation with mouse sera that had also been used in primary identification of the tested peptide antigens, 32 of the 48 peptides for which we anticipated reactivity, proved fully functional by displaying species-specific reactivity on the microarray (67%). This excluded two *C. pecorum* and two *C. trachomatis* strain-variant peptides from the total of 52 peptides evaluated. Seven more peptides were only marginally reactive giving signal intensities between 0.1 and 0.2 (14%), and nine were completely non-reactive (19%). However, 6 murine non-reactive peptides became functional when giving strong and repeated signals with sheep sera (Fig. 5) or human sera in Fig. 6A and B (Cfe-Fe/C_PmpD-1060-83, Cmu_Nigg3_CT618_186-209, Csu_99DC3_IncA_259-282, Ctr_D/UW-3_PmpD_540-563, Ctr_D/UW-3_PmpD_1039-1062, Cpn_CWL029_PmpD_1141-64). Therefore, altogether 45 of the 48 evaluated peptides (94%) proved to be functional.

The main cause of inefficient ELISA-to-microarray transfer for some peptide antigens is most likely the modification of the length of originally identified sequences (Supplementary Table S1). Two non-reactive peptides were substantially truncated, and one was elongated, and these three peptides never recovered reactivity in later versions of the microarray or by use of non-murine antisera. Another contributor to transfer inefficiency may be nucleophilic binding to the epoxy support^41^ by reactive amino acid side chains other than those of the N-terminal triple linker lysines. Such aberrant covalent linkage of peptide antigens may inhibit flexibility and mask antibody binding sites. When analyzing amino acids with reactive side chains, we found in non-transferred peptides a highly significant enrichment of lysine, methionine, and tyrosine and depletion of aspartic acid (P = 0.0003).

Two strain-variant peptides of *C. pecorum* (strains 66P130 and W73) did not react with *C. pecorum* E58-specific pooled mouse sera as expected due to high sequence divergence of strains 66P130 and W73 from E58. However, signals of the strain-variant peptides were seen in a number of bovine sera (Fig. 3), consistent with the exposure of the animals to multiple *C. pecorum* strains^37,38^. Two OmpA variable domain (VD)-I strain-variant peptides of *C. trachomatis* (Ctr_RC-J/953_OmpA_082-105, Ctr_F/SW4_OmpA_082-105) did not react with human sera, although OmpA VD-IV peptides of these strains showed reactivity (Fig. 6A). The reactivity of the OmpA VD IV peptides may be due to sequence homology with *C. trachomatis* serovar D, resulting in cross-reactivity (Fig. 1).

Compared to currently employed ELISA technology, several studies have demonstrated that the present ArrayTube platform works faster (approx. 2 h per run) and requires only minute amounts of serum, usually 1 μl of a 1:100 dilution^42–44^. More important, the information content of the experimental output is far higher, because reactive antibodies are characterized through their binding to a number of peptide antigens derived from multiple proteins. Thus, the serum sample can be described through its individual reaction pattern (antibody profile) rather than a simple positive-or-negative classification. The issue of individual serological response patterns or antibody profiles has been rarely addressed so far^45,46^, mainly because conventional serological tools are not suitable for their investigation.

It is this extended capacity of the peptide array that opens up new horizons in serological analysis and facilitates more detailed understanding of the humoral immune response at a molecular level. Using the present version of the microarray, we were able to identify and visualize individual antibody profiles of animals and humans. For instance, at least sheep 1 and 2 responded to vaccination with the *C. abortus* live vaccine in an individual fashion, i.e. showing different antibody levels and distinct signal patterns (Fig. 4). While vaccination raised *C. abortus*-specific antibody titres in these sheep, no response was seen with the inactivated vaccine. The *C. abortus*-specific antibodies were detected even when the animals had high antibody levels against other chlamydiae, in this case *C. pecorum*.

Individual differences in humoral response appeared as varying proportions of antibodies generated against each of the three *C. abortus* epitopes. Based on this molecular information, analysis of individual host responses will be possible in unprecedented detail. The fact that these sheep were carriers of *C. pecorum*, another potential pathogen, was completely missed by conventional serological tests, such as *C. abortus*-specific ELISA and CFT. Likewise, any *Chlamydiaceae*-specific ELISA would have failed to differentiate specific antibodies to each of the two chlamydial species in animals and humans alike. In the absence of a specific assay, evaluation of the effect of vaccination would be vague if not impossible.

The spectrum of species-specific chlamydial antibodies revealed by microarray can also be attributed to individual herds. According to the signal patterns observed, the two sheep herds apparently had different exposures to spurious, non-endemic chlamydial infections. While *C. pecorum* antibody levels are higher in herd 1 (Fig. 4, animals 1–3), one can speculate that herd 1 was additionally exposed to *C. pneumoniae*, and herd 2 (animals 4–9) to *C. muridarum* and *C. suis*, whereas both herds had some exposure to *C. psittaci* and *C. felis* through contact to carrier animals (Fig. 5).

Testing of the human samples revealed that correlation between microarray and ELISA results was far better for the *C. trachomatis*- positive and -negative sera compared to *C. pneumoniae* (Figs 6, and 7A,B). However, the reverse ROC analysis strongly suggested major problems with the ELISAs (Fig. 7C,D). The *C. trachomatis* ELISA was still acceptable, being characterized by poor sensitivity, but excellent specificity, relative to the *C. trachomatis* peptides in the microarray. In contrast, the performance of the *C. pneumoniae* ELISA resembles the roll of a dice, with 100% sensitivity, but an inacceptable specificity of 61%. This may have to do with well-known problems of *C. pneumoniae* serology (suboptimal assays, background of Cpn antibodies in a large fraction of the population, absence of humoral response in certain cases), but mainly with cross-reactivity of the *C. pneumoniae* ELISA antigen with antibodies against other *Chlamydia* spp. The ELISA antigen used in this study was the *C. pneumoniae* outer membrane complex (COMC) with its major constituent OmpA, which is not among the most immunodominant antigens^47,48^. In addition, chlamydial COMC is cross-reactive with antibodies against other *Chlamydia* spp., including *C. trachomatis*^47^. Thus, some of the numerous positives in the *C. pneumoniae* COMC ELISA may actually be false positives, thus creating the discrepant results with microarray.

The performance of the present assay reflects the degree of coverage of naturally relevant antigens and their epitopes that are represented on the peptide microarray. As can be seen from the collective data, the limited number of peptides representing *C. trachomatis, C. pecorum* and *C. abortus* that have been included here apparently sufficed for correct analysis of the sera. In a recent study, a combination of five peptides achieved 92% specificity and 94% sensitivity for detection of *C. trachomatis* antibodies (Rahman *et al*. unpublished data). Consequently, to ensure maximum sensitivity and specificity, the currently known peptide antigen repertoire of each chlamydial species^34^ should be extended to 5–7 peptide antigens from 3–5 proteins.

The data of this proof-of-concept study confirm the notion that factors like endemic dissemination of *Chlamydia* spp.^3^, multiple chlamydial infections in the same host^49,50^ and unnoticed latent infections^3,37,38,51^ are rendering serology of *Chlamydia* spp. infections a complex and methodologically challenging issue. The present microarray platform may well be suited to contribute to major advances in serology by allowing to obtain large datasets from a variety of hosts, either epidemic, naturally endemic, or transient. Future epidemiological studies will enable researchers and diagnosticians to reveal personalized antibody reaction patterns to host-specific epitopes and antigenic hallmarks of infective pathogen(s) and strain(s). An optimal assay for highly sensitive detection of chlamydial monospecies-specific IgG antibodies must include multiple peptide antigens from different immunogenic proteins, such as IncA, the Pmp family, OmpB, and type III secretion system components. Such a peptide microarray approach would be ideal to analyze the great variety of host antibody responses to any *Chlamydia* spp.

## Methods

### Hyperimmune sera from mice

Monospecific hyperimmune sera to strains B577 (C18/98) of *C. abortus*, 10DC88 of *C. avium*, GPIC (03DC25) of *C. caviae*, 02DC26 of *C. felis*, 08–1274/3 (08DC63) of *C. gallinacea*, MoPn (03DC39) of *C. muridarum*, E58 of *C. pecorum*, CDC/CWL-029 of *C. pneumoniae*, 03DC15 of *C. psittaci*, 99DC03 of *C. suis*, and D/UW-3/Cx of *C. trachomatis* were raised in Balb/c and A/J mice by 3 consecutive inoculations in a lung infection model as described previously^34^. Sera from 9–53 animals were pooled into a single batch for each *Chlamydia* spp.

### Sera from cattle with known history of *Chlamydia* infection

Three panels of bovine sera have been examined: (i) five cows that had experienced multiple episodes of natural infection with multiple *C. pecorum* strains^38^, (ii) five calves aged 11 to 15 weeks that had undergone a first episode of PCR-detected *C. pecorum* infection, and (iii) five seronegative calves aged 0 to 8 weeks without PCR-detected *C. pecorum* infection^37^.

### Sera from sheep with known *C. abortus* vaccination status

All ovine serum samples originated from 9 sheep that were vaccinated and then sampled three times in a previously described trial in Switzerland^52^. Briefly, animals 1, 2 and 3 (from herd 1) were vaccinated once intramuscularly using live vaccine Ovilis Enzovax™. In herd 2, sheep 4, 5 and 6 were vaccinated using the inactivated vaccine Ovax Clamidia, and sheep 7, 8 and 9 served as mock-vaccinated controls.

### Human sera with confirmed reactivity to *C. trachomatis* and *C. pneumoniae*

A total of 43 human samples have been included. Plasma samples 1–23 and 28–43 (Supplementary Table S4) were from plasma donations (plasmaphoresis) originated from the US and were collected in FDA-licensed and registered collection facilities (Commercial supplier: SLR Research Corporation, PO Box 2729, Carlsbad, CA 92018). Serum samples 24 and 25 were from blood donations collected in France (Établissement Français Du Sang- Normandie – 609, Chemin De La Bretéque, B.P. 558–76235 Bois Guillaume Cedex) and plasma samples 26 and 27 originated from blood donations collected in Germany (Blutspendedienst des Bayerischen Roten Kreuzes, gemeinnützige GmbH, Herzog-Heinrich Str. 2, 80336 München). Sample collection at all donation centers was conducted in accordance with national legislation, which included an informed consent scheme.

### Selection of peptide antigens for the microarray

The microarray peptide antigens were selected based on highly reactive, species-specific peptide antigens of chlamydial B-cell epitopes as described previously^33,34^. Briefly, one to five dominant polymorphic B-cell epitope regions had been identified on OmpA, CT618, PmpD, IncA, CT529, CT442, IncG, Omp2, TarP, and IncE proteins of all *Chlamydia* spp. Of these epitopes, peptide antigens with strong and specific reactivity with homologous, but not heterologous, *Chlamydia* monospecies-specific murine hyperimmune sera were selected for testing in the microarray format. For detection of antibodies against all known sero-variants, several strain-variant peptides were derived from the same epitopes of the *C. pecorum* IncA and *C. trachomatis* OmpA proteins.

### Preparation and production of the microarray

Microarray production and assay conditions were initially optimized for spacer and length of the peptide antigens, and for covalently linking the peptide antigens to the microarray epoxy surface. The final assay used the ArrayTube (AT) platform (Alere Technologies GmbH, Jena, Germany), which includes a 1.5-ml standard plastic reaction vial (Eppendorf type) equipped with a 3.4 × 3.4-mm three-dimensional epoxy-surface modified glass chip integrated in the bottom. The active surface area of 5.76 mm2 allows the placement of up to 207 peptide spots having an average diameter of 110 µm. Synthetic peptides as desalted extracts (peptides& elephants, Hennigsdorf, Germany) were spotted without any detergents at a final concentration of 0.5 mg/ml in phosphate-buffered saline (PBS) containing 20 mM trehalose. The technology includes a contact spotting procedure to exclude cross-contamination and carryover^53^. All peptides used consisted of three N-terminal lysine residues (KKK) coupled via trioxatridecan-succinamic acid (Ttds) with a twofold serine-glycine (SGSG) spacer followed by the specific chlamydial sequence and a C-terminal glycine.

For reasons of microarray production economy, specific chlamydial sequences of 16 to 24 amino acids were used. In comparison to the originally determined peptide antigens of 16–40 amino acids, 12 specific chlamydial sequences remained unchanged in length, 12 were extended to 20–24 amino acids, and the remaining 28 were shortened. Aside from spotted chlamydial peptides, each array contained control spots for staining and conjugate reaction (recombinant protein A/G-HRP), and background (spotting buffer). Each item was spotted two- or threefold in a randomized layout. The list of peptides with their aa sequences is given in Supplementary Table S1.

### Peptide microarray assay protocol

The Protein Binding Kit (Alere Technologies, Jena, Germany) was used according to the instructions of the manufacturer. All operations were conducted on an Eppendorf Thermomixer Comfort (Eppendorf, Hamburg, Germany) at 37 °C and shaking frequencies (rpm) as indicated. Briefly, each ArrayTube (AT) was conditioned by adding 500 µl binding buffer P1 and shaking at 400 rpm for 5 min, and subsequently blocked by treatment with 100 µl blocking buffer (2% milk powder (Roth, Karlsruhe, Germany) in buffer P1) at 300 rpm for 5 min. After removal of liquids, 100 µl of a 1:100 dilution in P1 of the serum sample was incubated in the AT vessel upon 300 rpm for 30 min. This reaction step was followed by the first wash step (500 µl P1, 400 rpm, 5 min), conjugation (100 µl of 1:5,000 diluted Protein A/G-HRP or anti-human-IgG-HRP (Sigma), 300 rpm, 30 min), and the second wash step (as above). Reactive spots were visualized by incubating the AT with 100 µl of HRP substrate D1 at ambient temperature without shaking for 10 min. Stained microarrays were processed using the Iconoclust software, version 3.3 (Alere) to yield numerical output as relative spot intensities (RSI), i.e. normalized background-corrected spot intensity values covering the range from 0 (no signal) to 1 (maximum signal), and graphical output as bar diagrams.

### Validation of seroreactivity of microarray-spotted peptide antigens

The seroreactivity of the selected chlamydial peptide antigens was evaluated by use of different sets of animal and human sera as described above. The presence of specific anti-chlamydial IgG antibodies in these sera had been characterized by different antibody assays. Sera from mice were examined using a peptide-based chemiluminescent ELISA^34^. Cattle sera were tested with chemiluminescent ELISAs based on peptide antigens^34^ and on antigens of *C. pecorum* elementary body lysates^37,38^. Sheep sera were characterized using a competitive ELISA including a *C. abortus*-specific monoclonal antibody^54^ and complement fixation test^39^. The panel of human serum and plasma samples was characterized using species-specific ELISAs for antibodies against *C. trachomatis* (SERION ELISA classic Chlamydia trachomatis IgG, ESR 1372 G, Institut Virion\Serion GmbH, Würzburg, Germany) and *C. pneumoniae* (SERION ELISA classic Chlamydia pneumoniae IgG, ESR 1371 G, Institut Virion\Serion GmbH, Würzburg, Germany). The *C. trachomatis* ELISA is based on a recombinant major outer membrane protein (MOMP) antigen, and the *C. pneumoniae* ELISA uses chlamydial outer membrane protein complexes (COMC) as antigen.

### Statistical analysis

Statistical analyses were performed and graphical outputs were generated by Microsoft Excel 2016, the software package Statistica 7.1 (Statsoft, Tulsa, Oklahoma, USA), and freeware JavaScript programs JROCFIT and JLABROC4 for calculating receiver operating characteristic (ROC) curves (Reference: Eng J. ROC analysis: web-based calculator for ROC curves. Baltimore: Johns Hopkins University [updated March 19; 2014; cited *Jan 4, 2017*], available from: http://www.jrocfit.org). For determination of relationships between continuous variables, Pearson’s correlation coefficients (R) were calculated in linear and polynomial regression analyses from R-square, and bivariate scatterplots of the variables were generated. R values of 0.01–0.30 were considered as very weak, 0.30–0.50 as weak, 0.50–0.70 as moderate, 0.70–0.90 as strong, and 0.90–1.00 as very strong correlation. The non-parametric Mann-Whitney U test was used to compare average anti-*C. pecorum* antibody levels in cattle with low exposure to *C. pecorum* versus cattle with high exposure to *C. pecorum*. Reaction frequencies of sheep sera with *C. abortus* peptides before and after *C. abortus* vaccination were analyzed using Fisher exact test, and two-sided probabilities were reported. Binormal ROC curves were constructed by maximum likelihood estimation from continuously distributed maximal microarray RSI data or from ordinal ELISA scores.

### Data availability

All data generated and analyzed during this study are included in this published article and its Supplementary Information files.

## Electronic supplementary material

Supplementary Dataset
