# Supplementary material for: A novel synthetic peptide microarray assay detects Chlamydia species-specific antibodies in animal and human sera
Source: Sci Rep. 2018 Mar 16;8:4701. doi: 10.1038/s41598-018-23118-7 (PMC5856796; doi:10.1038/s41598-018-23118-7)
Supplement: Supplementary file 1 — Supplementary Dataset [file 41598_2018_23118_MOESM1_ESM.zip › Supplementary_items.pdf]

A novel synthetic peptide microarray assay detects *Chlamydia* species-specific antibodies in animal and human sera

Konrad Sachse[1]*, Kh. Shamsur Rahman[2], Christiane Schnee[1], Elke Müller[3,4], Madlen Peisker[1], Thomas Schumacher[5], Evelyn Schubert[1], Anke Ruettger[1], Bernhard Kaltenboeck[2], Ralf Ehricht[3,4]

[1] Institute of Molecular Pathogenesis, Friedrich-Loeffler-Institut (Federal Research Institute for Animal Health), Jena, Germany
[2] Department of Pathobiology, College of Veterinary Medicine, Auburn University, Auburn, AL (USA)
[3] Alere Technologies GmbH, Jena, Germany
[4] InfectoGnostics Research Campus, Jena, Germany
[5] Institut Virion\Serion GmbH, Würzburg, Germany

***Corresponding author**: Dr. Konrad Sachse, Institute of Molecular Pathogenesis, Friedrich-Loeffler-Institut (Federal Research Institute for Animal Health), Naumburger Str. 96a, 07743 Jena, Germany
Present address: RNA Bioinformatics and High-Throughput Analysis, Faculty of Mathematics and Computer Science, Friedrich-Schiller-Universität, Leutragraben 1, 07743 Jena, Germany, e-mail konrad.sachse@uni-jena.de

Supplementary Information

Supplementary Table S1: Amino acid sequences of peptides used on the microarray. Shared amino acids between actual microarray peptides and the originally identified epitopes are in red font.

SN	Peptide	Microarray peptide sequence	Rahman et al, 2015	Original Rahman peptide sequence
1	Cab_S26/3_OmpA_158-173	GVKGSSIAADQLPNV	Cab_S26/3_OmpA_153-176	NLVGLIGVKGSSIAADQLPNVGIT
2	Cab_S26/3_OmpA_153-176	NLVGLIGVKGSSIAADQLPNVGIT	Cab_S26/3_OmpA_153-176	NLVGLIGVKGSSIAADQLPNVGIT
3	Cab_S26/3_IncA_328-351	TEHADIPRDPNRDPRGGRGGQSSP	Cab_S26/3_IncA_324-353	STAVTEHADIPRDPNRDPRGGRGGQSSPSV
4	Cab_S26/3_PmpD_1064-1087	PTSNVYYSAHESVKQPENKTLADI	Cab_S26/3_PmpD_1060-1089	KIESPTSNVYYSAHESVKQPENKTLADINS
5	Cps_02DC15_OmpA_158-181	LVGLIGFSAASSISTDLPTQLPNV	Cps_02DC15_OmpA_158-181	LVGLIGFSAASSISTDLPTQLPNV
6	Cps_02DC15_OmpA_329-344	SLIGSTTALPNNSGKD	Cps_02DC15_OmpA_333-348	STTALPNNSGKDVLSD
7	Cps_02DC15_OmpA_329-352	SLIGSTTALPNNSGKDVLSDVLQI	Cps_02DC15_OmpA_333-348	STTALPNNSGKDVLSD
8	Cps_02DC15_IncA_329-352	ADQGDLRDPSGDRYGGWGAQSSYR	Cps_02DC15_IncA_321-360	SLTSTTETADQGDLRDPSGDRYGGWGAQSSYRLSPSVTMS
9	Cps_02DC15_CT618_108-131	DSATGSFKIVTKNIQKPNGEVEIV	Cps_02DC15_CT618_105-134	YEVDSATGSFKIVTKNIQKPNGEVEIVSSR
10	Cps_02DC15_CT618_198-221	IVSTLRSTDFDPSYEDLVQRRVTL	Cps_02DC15_CT618_189-228	CGAVDDVISIVSTLRSTDFDPSYEDLVQRRVTLREKFFSL
11	Cca_GPIC_OmpA_157-176	LGVTGTDLQGQYPNVAISQG	Cca_GPIC_OmpA_159-168	VTGTDLQGQY
12	Cca_GPIC_IncA_332-355	EASQDDSAQPQDENQSDAGEHKDS	Cca_GPIC_IncA_316-355	LIGVMVQDGAESSTVEEASQDDSAQPQDENQSDAGEHKDS
13	Cca_GPIC_CT618_140-163	GNFVLQTKTVQLEDGTQRVVPSRV	Cca_GPIC_CT618_134-163	EVDAQTGNFVLQTKTVQLEDGTQRVVPSRV
14	Cca_GPIC_CT618_225-248	DIVSLVRSPTGDLSTEELSERRQT	Cca_GPIC_CT618_220-249	ADDAVDIVSLVRSPTGDLSTEELSERRQTL
15	Cfe_Fe/C_OmpA_160-175	IGLAGTDFANQRPNVE	Cfe_Fe/C_OmpA_160-175	IGLAGTDFANQRPNVE
16	Cfe_Fe/C_OmpA_160-179	IGLAGTDFANQRPNVEISQG	Cfe_Fe/C_OmpA_160-175	IGLAGTDFANQRPNVE
17	Cfe_Fe/C_PmpD_1060-1083	VEKIESPSAKSYYSNYEIEKNPIE	Cfe_Fe/C_PmpD_1055-1084	PNVKSVEKIESPSAKSYYSNYEIEKNPIEK
18	Cfe_Fe/C_CT618_113-136	NFKIGVKSVKNENGETVLVPCRIL	Cfe_Fe/C_CT618_108-137	DSASGNFKIGVKSVKNENGETVLVPCRILK
19	Cav_10DC88_IncA_305-328	ESTPVEAPESKEEAKDTAEVAAEG	Cav_10DC88_IncA_299-328	TVEGAAESTPVEAPESKEEAKDTAEVAAEG
20	Cav_10DC88_IncA_325-348	AAEGSGSTEESKGKEDDKSGDKKE	Cav_10DC88_IncA_319-348	KDTAEVAAEGSGSTEESKGKEDDKSGDKKE
21	Cga_08DC63_OmpA_326-345	NPSFLGSADAQATLVDSVQI	Cga_08-1274/3_OmpA_326-345	NPSFLGSADAQATLVDSVQI
22	Cga_08DC63_IncA_303-326	TSTPEGETSETKEGEEDSSVVEFD	Cga_08-1274/3_IncA_297-326	SEAATSTSTPEGETSETKEGEEDSSVVEFD
23	Cpe_E58_CT442_167-190	PGSGGSGGVSASGALEQVANIVMN	Cpe_E58_CT442_151-190	DGSNQIFVDSNRDIRRPGSGGSGGVSASGALEQVANIVMN
24	Cpe_E58_CT529_217-240	QRCLERLNQKEVGQEESGSAQEVQ	Cpe_E58_CT529_209-248	IIRERRAYQRCLERLNQKEVGQEESGSAQEVQAMRSSYVK
25	Cpe_E58_IncA_273-296	QQAAAPAAPAAPAAPAAPAAPAAP	Cpe_E58_IncA_281-300	AAPAAPAAPAAPAAPA
26	Cpe_E58_IncA_311-326	PAPENNDNNNDDNAAS	Cpe_E58_IncA_311-326	PAPENNDNNNDDNAAS
27	Cpe_E58_IncA_303-326	AAPAAPAAPAPENNDNNNDDNAAS	Cpe_E58_IncA_311-326	PAPENNDNNNDDNAAS
28	Cpe_3257_IncA_303-326	IHAAAPAAPAAPENNDNNDDNAAS	Cpe_E58_IncA_311-326	PAAPENNDNNDDNAAS
29	Cpe_PV3056_IncA_303-326	APAAPAAPENNDNNNDNNDDDAAS	Cpe_E58_IncA_311-326	ENNDNNNDNNDDDAAS
30	Cpe_66P130_IncA_303-326	AAGAAGAAGAAGAAGENQNEGDEG	Cpe_E58_IncA_311-326	GAAGAAGENQNEGDEG
31	Cpe_W73_IncA_278-301	APEAPAPEAPAPEAPAPEAPAPEN	Cpe_E58_IncA_311-326	APAPEAPAPEAPAPEN
32	Cpn_CWL029_IncA_331-360	ESEFIACVRDRTFGRRETPPPTTP	Cpn_CWL029_IncA_331-370	QKAESEFIACVRDRTFGRRETPPPTTPVVEGDESQEEDEG
33	Cpn_CWL029_IncA_336-359	EFIACVRDRTFGRRETPPPTTPVV	Cpn_CWL029_IncA_331-370	QKAESEFIACVRDRTFGRRETPPPTTPVVEGDESQEEDEG
34	Cpn_CWL029_PmpD_1141-1164	VQINMSSPTPNKDKAVDTPVLADI	Cpn_CWL029_PmpD_1131-1170	NKEETLVSAGVQINMSSPTPNKDKAVDTPVLADIISITVD
35	Cpn_CWL029_CT618_201-216	PETISDPENRNKPSAE	Cpn_CWL029_CT618_201-216	PETISDPENRNKPSAE
36	Cmu_Nigg_PmpD_719-742	EENEEKVETADINSDKQEAEERSL	Cmu_Nigg_PmpD_724-739	KVETADINSDKQEAEE
37	Cmu_Nigg3_PmpD_1033-1056	QENNAEIGDLEDSVNSEKTPSLWI	Cmu_Nigg_PmpD_1038-1053	EIGDLEDSVNSEKTPS
38	Cmu_Nigg3_CT618_186-209	ATLTTKDKCCDSTQGNSFMERFKR	Cmu_Nigg_CT618_190-205	TKDKCCDSTQGNSFME
39	Cmu_Nigg3_CT618_190-205	TKDKCCDSTQGNSFME	Cmu_Nigg_CT618_190-205	TKDKCCDSTQGNSFME

40	Csu_99DC3_CT529_213-236	ILCGQADEVLGINNTMCEQFVRQR	Csu_99DC3_CT529_207-236	EERCNRILCGQADEVLGINNTMCEQFVRQR
41	Csu_99DC3_IncA_259-282	LANIKEALIKPSRPPLPKNGFPRT	Csu_99DC3_IncA_259-293	LANIKEALIKPSRPPLPKNGFPRTMPPCPPRQTPP
42	Ctr_D/UW-3_OmpA_082-105	FQMGAKPTTDTGNSAAPSTLTARE	Ctr_D/UW-3_OmpA_082-105	FQMGAKPTTDTGNSAAPSTLTARE
43	Ctr_RC-J/953_OmpA_082-105	FQMGAAPTTSDVAGLQNDPTTNV	Ctr_D/UW-3_OmpA_082-105	FQMGAAPTTSDVAGLQNDPTTNV
44	Ctr_F/SW4_OmpA_082-105	FEMGEALAGASGNTTSTLSKLVER	Ctr_D/UW-3_OmpA_082-105	FEMGEALAGASGNTTSTLSKLVER
45	Ctr_D/UW-3_OmpA_313-328	IFDTTTLNPTIAGAGD	Ctr_D/UW-3_OmpA_313-328	IFDTTTLNPTIAGAGD
46	Ctr_D/UW-3_OmpA_313-336	IFDTTTLNPTIAGAGDVKTGAEGQ	Ctr_D/UW-3_OmpA_306-345	QPKSATAIFDTTTLNPTIAGAGDVKTGAEGQLGDTMQIVS
47	Ctr_RC-J/953_OmpA_313-336	ILDVTTLNPTIAGKGTVVASGSEN	Ctr_D/UW-3_OmpA_306-345	QPKLAEAILDVTTLNPTIAGKGTVVASGSENDLADTMQIVS
48	Ctr_F/SW4_OmpA_313-336	VVDITTLNPTIAGCGSVAGANTEG	Ctr_D/UW-3_OmpA_306-345	QPRLVTPVVDITTLNPTIAGCGSVAGANTEGQISDTMQIVS
49	Ctr_D/UW-3_CT529_208-231	ARCARIAREESLLEVPGEENACEK	Ctr_D/UW-3_CT529_200-239	SAERADCEARCARIAREESLLEVPGEENACEKKVAGEKAK
50	Ctr_D/UW-3_PmpD_540-563	QALPTQEEFPLFSKKEGRPLSSGY	Ctr_D/UW-3_PmpD_536-565	ARAPQALPTQEEFPLFSKKEGRPLSSGYSG
51	Ctr_D/UW-3_PmpD_1039-1062	PVQQGHAISKPEAEIESSSEPEGA	Ctr_D/UW-3_PmpD_1036-1065	SGTPVQQGHAISKPEAEIESSSEPEGAHSL
52	Ctr_D/UW-3_IncG_123-146	SASPQASPTSSTFGLESALRSIGD	Ctr_D/UW-3_IncG_108-147	RPSDQQESGGRLSEESASPQASPTSSTFGLESALRSIGDS

Color scale is the same as in Fig. 1

Supplementary Table S2 Examination of field sera from cattle with known infection history using the peptide microarray assay

Peptide ID	Reactivity of *Chlamydia pecorum* peptides with serum antibodies														
	1	2	3	4	5	6	7	8	9	10	cow 1	cow 2	cow 3	cow 4	cow 5
Cpe_E58_IncA_273-296				0.12	0.26		0.66		0.75	0.75	0.26			0.37	
Cpe_W73_IncA_278-301				0.33	0.37	0.63		0.57	0.51	0.59	0.29				0.13
Cpe_E58_IncA_303-326		0.12	0.13		0.11		0.42		0.72	0.40	0.61	0.76		0.51	0.14
Cpe_PV3056_IncA_303-326		0.17					0.40		0.68	0.40	0.61	0.69		0.63	
Cpe_3257_IncA_303-326		0.38	0.15	0.13	0.29		0.43		0.69	0.64	0.67	0.72	0.11	0.66	0.36
Cpe_66P130_IncA_303-326						0.20		0.68	0.55	0.13	0.40		0.70	0.74	0.67
Cpe_E58_CT529_217-240							0.42								
Cpe_E58_CT442_167-190							0.27								
Other reactions				Cga 0.38	Cga 0.50		Ctr 0.19 0.21<br>Cav 0.49		Cmu 0.20	Cmu 0.20	Cpn 0.60<br>Csu 0.33		Ctr 0.22	Cab 0.34<br>Cfe 0.60	
ELISA	neg	neg	neg	neg	neg	pos	pos	pos	pos	pos	pos	pos	pos	pos	pos

Numerical values represent signal intensity: max 1.0, min 0, cut-off 0.1 (average signal intensity of 3 spots)

Supplementary Table S3 Examination of sera from sheep of known vaccination status

Animal	dpv (vaccine)	CFT	ELISA[a]	Peptide Array		
				C. abortus[b]	*C. pecorum*[b]	Other reactions
1	0 (LVac)	-ve	-ve	0	0.21-0.81 (5/8)	
2	0 (LVac)	-ve	-ve	0	0.28-0.76 (5/8)	Cpn 1/3
3	0 (LVac)	-ve	-ve	0	0	
1	7	-ve	-ve	0	0.36-0.72 (5/8)	
2	7	-ve	-ve	0	0.27-0.60 (4/8)	Cpn 1/3
3	7	-ve	borderline	0	0.11 (1/8)	
1	21	**1:320** +++	**+ve**	**0.17-0.30** (3/3)	0.42-0.84 (5/8)	Cfe 2/3, Cav 1/7
2	21	**1:80** ++++	**+ve**	**0.32-0.56** (3/3)	0.17-0.73 (5/8)	Cps 1/5, Ctr 1/11
3	21	**1:40** ++++	**+ve**	**0.12** (1/3)	0.28 (1/8)	Cfe 1/3
4	0 (iVac)	-ve	-ve	0	0	
5	1 (iVac)	-ve	-ve	0	0	
6	2 (iVac)	-ve	-ve	0	0	
4	14	-ve	-ve	0	0	
5	14	-ve	-ve	0	0	
6	14	-ve	-ve	0	0	
4	28	-ve	-ve	0	0	
5	28	-ve	-ve	0	0	
6	28	-ve	-ve	0	0	
7	0 (control)	-ve	-ve	0	0	
8	0 (control)	-ve	-ve	0	0.21 (1/8)	Cmu 1/3
9	0 (control)	-ve	-ve	0	0.21-0.27 (2/8)	Cfe 1/3
7	14	-ve	-ve	0	0	
8	14	-ve	-ve	0	0.24 (1/8)	Cps 1/5, Cmu 1/3
9	14	-ve	-ve	0	0.24-0.51 (3/8)	Cfe 1/3, Csu 1/3
7	28	-ve	-ve	0	0	
8	28	-ve	-ve	0	0.12 (1/8)	Cps1/5, Cmu 1/3
9	28	-ve	-ve	0	0.28 (1/8)	Csu 1/2

Legend to Table S3

dpv days post vaccination, LVac live vaccine *C. abortus*, iVac inactivated vaccine *C. abortus*

[a] Competitive ELISA using *C. abortus*-specific monoclonal antibody (reference 30)

[b] Numerical values represent signal intensity: max 1.0, min 0, cut-off 0.1 (average intensity of 3 spots); in brackets: number of reacting peptides out of the total number covering the respective species on the microarray

Supplementary Table S4 Examination of human sera

Sample # (Fig.6)	ELISA Ctr/Cpn	Peptide Microarray No. of reactive peptides (species)	Signal intensity range
1	++++/-	5/11 (Ctr)	0.31-0.82
2	++++/-	6/11 (Ctr)	0.44-0.82
3	++++/-	4/11 (Ctr)	0.77-0.80
4	++++/+	2/11 (Ctr)	0.45-0.79
5	++++/-	4/11 (Ctr)	0.71-0.76
13	++++/-	0	0
6	+++/++	7/11 (Ctr)	0.13-0.74
7	+++/+++	5/11 (Ctr)	0.10-0.74
8	+++/+++	2/11 (Ctr)	0.39-0.68
9	+++/+++	5/11 (Ctr)	0.13-0.53
14	++/+	5/11 (Ctr), 1/4 (Cpn)	0.16-0.56
19	++/+++	3/11 (Ctr), 2/4 (Cpn)	0.11-0.61
16	++/+++	2/11 (Ctr), 1/4 (Cpn)	0.18-0.47
17	++/++	3/11 (Ctr)	0.12-0.34
11	+/+++	3/11 (Ctr)	0.12-0.50
20	+/+++	1/11 (Ctr), 2/4 (Cpn)	0.10-0.46
21	+/+++	3/11 (Ctr)	0.10-0.36
24	+/+++	2/11 (Ctr), 2/4 (Cpn)	0.13-0.36
25	+/+++	2/11 (Ctr), 1/4 (Cpn)	0.10-0.21
23	+/++++	4/11 (Ctr), 1/4 (Cpn)	0.10-0.21
26	+/+++	3/4 (Cpn)	0.24-0.79
27	+/+++	0	0
28	+/+++	0	0
30	+/+++	0	0
36	+/+++	2/4 (Cpn)	0.25-0.71
18	+/+	0	0
42	+/+	0	0
43	+/+	0	0
10	-/++++	4/11 (Ctr)	0.38-0.52
12	-/++++	3/11 (Ctr)	0.11-0.41
15	-/++++	6/11 (Ctr), 2/4 (Cpn)	0.14-0.56
22	-/++++	3/11 (Ctr), 1/4 (Cpn)	0.14-0.20
32	-/++++	1/4 (Cpn)	0.49
29	-/-	0	0
31	-/-	0	0
33	-/-	1 (Cpn)	0.14
34	-/-	0	0
35	-/-	0	0
37	-/-	0	0
38	-/-	0	0
39	-/-	1/4 (Cpn)	0.16
40	-/-	0	0
41	-/-	0	0

ELISA scale: **-** ... no signal, + ... weak signal below cut-off, ++... borderline, +++... positive, ++++... strongly positive
